# The Hospital. Nursing Section

**Published:** 1901-11-23

**Authors:** 


### The Hospital.

### Nursing Section.

Contributions for this Section of "The Hospital" should be addressed to the Editor, "The Hospital" Nursing Section, 28 & 29 Southampton Street, Strand, London, W.C.

No. 791.—Vol. XXXI. *SATURDAY, NOVEMBER* 23, 1901.

### Notes on News from the Nursing World.

### THE PENSION FUND SOUVENIR.

As the proprietors of The Hospital have received many inquiries, they desire to state that any reader who desires to do so, may purchase a copy of the souvenir and the plate, but those who do not order at once may be disappointed as only a limited number will be published. Order forms have been sent to all members of the Royal National Pension Fund, but owing to the frequent movements of nurses, all may not reach those addressed. In this case the proprietors of The Hospital will be glad to hear from any member who has not received an order form, so that one may be sent. Another point of inquiry is the size of the plate. The picture itself is 12 inches high by 15½ wide; on the mount it measures 18 high by 21 wide. The size of the souvenir album is 13 inches by 10 inches. The cover is in grey and tied with a red ribbon lettered in black and white.

### THE ALEXANDRA NURSES.

To the announcement that the appeal issued by the Queen, as Princess of Wales, on behalf of the wives and families of soldiers and sailors will henceforth be called "Queen Alexandra's Appeal," it may be added that the nurses employed under the auspices of the Soldiers and Sailors' Families' Association will continue to be called "Alexandra Nurses." The attention given by her Majesty to other organisations, and the creation of Queen Alexandra's Imperial Military Nursing Service, does not in any way diminish her interest in the branch of the Soldiers and Sailors' Families' Association, which, by special request, was called by her name.

### THE MATRON-IN-CHIEF OF THE QUEEN'S MILITARY NURSING SERVICE.

Although nurses are interested, in a general way, in the constitution of the Advisory Board for the supervision of Army medical services, the nomination about which they are most concerned has not yet been announced. Upon the choice of the matron-in-chief of Queen Alexandra's Imperial Military Nursing Service "to be nominated hereafter," much will depend; and if the civilian members of the Advisory Board have any voice in it, we may be sure that such men as Dr. Perry, Sir Frederick Treves, and Mr. Alfred Fripp will not fail to recommend the selection of a lady who is thoroughly in touch with the nursing world. Several more or less eligible individuals are mentioned in connection with the post, but it is to be hoped that practical experience, personal standing, and proved capacity will be the only bases for the nomination.

### MATRONS FOR THE CONCENTRATION CAMPS.

The matron of Guy's Hospital has received the following letter from the Under-Secretary of State for the Colonies: "I am directed by Mr. Secretary Chamberlain to acknowledge the receipt of your letter of the 11th instant, recommending Miss A. Willes, Miss C. R. Jones, Miss S. A. Hyland, and Miss E. K. Finnemore for appointments as matrons in the concentration camps in the Transvaal. Mr. Chamberlain has offered appointments to these ladies, subject to their being passed by the medical adviser of this department as physically fit for service, and he desires me to express to you his sincere thanks for the trouble which you have taken in selecting candidates."

### THE WAR NURSES.

The *Manchester Merchant* arrived at Southampton on Monday with Sisters F. M. Morris and C. Erichsen on board. Sister Morris (A.N.S.R.) requires two months' leave, resigns army duty, and wishes return passage to South Africa. Sister Erichsen (civil nurse, Sweden) is time-expired. The *Orient*, with Nursing Sisters J. M. Clay, L. Allman, and A. F. Clarke on board, is due at Southampton December 3; and the *Aurania*, with Nursing Sisters Chadfield, Audrey, and Newbold on board, December 8. Nursing Sister E. M. Brett, Johannesburg, is progressing favourably, but Nursing Sister Elizabeth Marshall is reported dangerously ill at Standerton.

### THE CASE OF AN EX-RESERVE SISTER.

Our attention has been called to an article in a weekly contemporary enlarging on the alleged hard case of a member of the Army Nursing Service Reserve whose services have been dispensed with by the War Office. We have reason to believe that the whole of the circumstances received the most careful attention of the authorities, and that they came to the conclusion that no other course was open to them. It may be added that if the nurse in question considers it worth while, she will doubtless be able to induce Mr. Labouchere, or some other member of Parliament, to bring the case before the House of Commons when the vote for the Army Medical Department is discussed, and the facts can be ascertained in the usual way.

### IN MEMORIAM.

It will be learnt with interest by our readers that the nursing sisters who are serving, and have served, during the present campaign in South Africa have subscribed and handed over to the treasurer of the Cape Town Cathedral Memorial Fund the sum of £300 for the purpose of placing in the new cathedral a stained-glass window to the memory of those sisters who have laid down their lives in the performance of their duty. The amount is handsome, and the mode of commemorating the devotion of the nurses who sacrificed themselves for the sake of ministering to the necessities of the defenders of their country has been most admirably chosen.

### NURSING IN IRISH WORKHOUSE INFIRMARIES.

At a conference held in Belfast under the auspices of the Irish Workhouse Association, an interesting paper on "Nursing in Workhouse Infirmaries and Hospitals" was read by Dr. Hall, who, in conclusion, said he felt bound, as a workhouse medical officer, to give full credit to the Local Government Board for Ireland and their inspectors for the valuable assistance and encouragement they have given in the endeavours to raise the status of the workhouse nurses. Referring to the recent Order, Dr. Hall said it was a matter for congratulation that the nurses are now "entirely removed from the control of the workhouse matron, the master of the workhouse being allowed disciplinary control." Dr. Hall evidently does not think that the disciplinary control should be maintained, for he went on to observe that he "saw no reason why the superintendents should not be able to allow the nurses to leave their infirmaries without having to request permission from the workhouse authorities." In other words, he would have infirmaries governed as ordinary hospitals. That is, of course, the standard to aim at, but at present England is in one respect behind Ireland, since the infirmary nurses on this side of St. George's Channel are not yet all entirely removed from the control of the workhouse matron. We are glad to see that even those who admittedly attended the conference at Belfast for the purpose of cursing Dr. Hall's views remained to bless them. He has done something towards reconciling angry guardians to the Order which, by slow degrees, they are learning to understand is mandatory and cannot be disregarded. The Armagh guardians do, indeed, continue to raise the standard of revolt, and have again declined to appoint a qualified nurse. But they will have to give way.

### NUNS AS NURSES.

The upshot of a long and friendly correspondence between some of the Irish Roman Catholic bishops and the Local Government Board of Ireland respecting the qualifications required of nuns for appointment as assistant nurses in workhouse hospitals is that the former propose that a certificate given by the nun in charge of a workhouse hospital, to the effect "that Sister X has gone through, say, a period of training for two years, and is competent to take charge of a workhouse hospital," should be accepted by the Board as sufficient. In most courteous language, however, the Vice-President of the Board closes the correspondence with the suggestion that the certificates should be signed by qualified medical men. The suggestion may, we conclude, be regarded as an ultimatum; and though the bishops have objected, on behalf of the nuns, to a doctor's certificate being required, they will, doubtless, recognise on further consideration that it cannot be dispensed with.

### FRICTION AT A CAPETOWN HOSPITAL.

It appears that friction among the nursing staff is not confined to workhouse infirmaries in the United Kingdom. At the Somerset Hospital, Capetown, the appointment of a committee to inquire into the difficulties which have arisen, and to take evidence, has been appointed. It is conceivable that they have to do with the nursing accommodation, which is notoriously inadequate. The Hospital Board recognised the need of new nurses' quarters some time since by inviting designs for a building. But delay has arisen owing to the fact that the only satisfactory designs sent in largely exceed the amount which the board have set aside for the purpose. This amount is £10,000. The board have now decided that the erection and completion of a suitable home will cost £14,000, and they will not proceed at all with the work until they have £12,000 in hand. We do not say that their policy is unsound, but we have no doubt that they will find it easier to prevent friction among the members of the nursing staff when they have made ample provision for their requirements.

### SHUTTING THE DOOR AGAINST THE NURSE.

The Chairman of Riddings Nursing Association is responsible for the statement that the people in this Derbyshire parish "shut the doors of their houses against the nurse"; and as the nurse herself has resigned on the ground of insufficiency of work, it must be concluded that her services have been deliberately refused. In this instance there was no lack of financial support. On the contrary, the balance in hand is £43. The result of a long discussion respecting the position was that it was decided to use the money in hand "to provide a local nurse under the direction of the doctors attending and a more skilled nurse when necessary, an executive committee to be elected to inquire into the same." It is curious that a resolution in favour of this action was moved by the Rev. A. Cotton, who had previously urged that "there were people in the village well able to nurse and to help in the household in a general way." If Mr. Cotton has been expressing these views among the poor and ignorant in Riddings, the fact that they shut the door against the trained nurse who does not help in the household "in a general way" may be partly accounted for.

### THE DIETARY SCALE FOR NIGHT NURSES.

In response to the suggestion of a matron, that the dietary scale for night nurses of some of the hospitals should be published, the medical officer of the Isolation Hospital, Beddington Corner, in charge of the Croydon Rural District Council, supplies us with the following information:—The nurses on night duty are allowed for consumption each night, in addition to the ordinary diet scale, an ounce of tea, an ounce of butter, half a pound of bread, a quarter of a pint of milk, an ounce of sugar, and either two eggs, three-ounce rasher of bacon, or half a pound of dried fish. The ordinary scale is very liberal, and includes per week 10½ lbs. of meat, 10½ lbs. of bread, 7 lbs. of potatoes, 2s. worth of other vegetables, a pint of jam, 1¼ lb. of bacon, 14 eggs, 7 pints of milk, and 1 lb. of tea, coffee, or cocoa.

### THE UNIFORM QUESTION AT DUNDEE.

A point of some interest has been raised in connection with the question of the new uniform ordered at Dundee Parochial Hospital by the authorities. It is the custom of the latter when they advertise for nurses to offer a salary of £35 a year "and uniform." The uniform, therefore, in the opinion of the nurses, becomes their own; and for this reason two of them refused to return the old dresses when new were given to them, the latter, it is alleged, being six months overdue. Failing a special agreement, the nurses are mistaken on the point of ownership, and, as, in any case, it is admitted that the old dresses would not have sold for sixpence, we are rather surprised both that the authorities should have required them to be returned, and that any of the nurses should have thought it worth while to refuse compliance.

### THE "NURSE'S NEVER."

Several correspondents have sent us letters of protest respecting the advice given under the heading of the "Nurse's Never" by a medical lecturer on nursing, and some of them urge that it can only apply to probationers. Possibly the author, who alone is responsible for his admonitions, had probationers only in mind, though as "G. M." shows in her letter this week, fully-trained nurses are sometimes guilty of at least one of the acts of forgetfulness he indicates. As it is possible he may be expected to take some notice of objections, we may say that since he fills a prominent position in connection with a leading hospital at the Antipodes, there may be apparent delay in answering them. It is due to him to add that his hospital experience is not confined to Australia. He has also been attached to the residential staff of a well-known hospital in Scotland.

### IMPROVEMENTS AT NEWTON ABBOT INFIRMARY.

The Newton Abbot Guardians have decided to adopt the recommendation of the medical officer to appoint two additional night nurses. The patients will now receive the same attention by night as by day. Dr. Ley, in the discussion on the subject, considered the additional nurses unnecessary, and contended, although he did not move an amendment, that there was not a workhouse infirmary in England with four wards and four fully-paid night nurses. But the visiting committee were unanimous in recommending the change, and were strongly backed up by Dr. Culver. We are glad to learn that the guardians contemplate the transformation of the existing maternity ward into a home for the nurses, and the construction of a new maternity block at the back of the infirmary.

### DUBLIN NURSES' CLUB.

The first annual meeting of the Dublin Nurses' Club was held last week. The president, Miss Huxley, gave a gratifying account of the progress of the club. During the year about 4,000 visits were paid by the members, who number over 500, and they introduced more than 300 visitors. The financial statement submitted was satisfactory, the accounts closing with a good balance, showing the appreciation with which the movement has been received by the nurses. Miss MacDonnell was elected vice-president for the ensuing year, and in order to develope the work and deepen the interest of the members, three sub-committees were formed to arrange for lectures, provide a library, and organise social evenings.

### A SERIOUS DEFICIT AT BRIGHTON.

The fact that at the annual meeting of the Brighton, Hove and Preston Nursing Association Mr. R. Y. Bevan had to announce that a balance of £462 was due to the treasurer is very discreditable to the people of Brighton. Except the statement that only one night nurse is employed by the Association, owing to lack of support, the report of the work is highly satisfactory. During the last 12 months 1,164 cases were nursed, and nearly 37,000 visits were paid. Mr. Gerald Loder, M.P., who in his excellent address did not mince his words as to the duty of the public, pointed out that the visits paid by the nurses cost, at the most, 9d. each. It cannot therefore be pretended that there is any extravagance in the administration of the funds. The Inspector of Nursing to Queen Victoria's Jubilee Institute has also spoken in favourable terms of the character of the training which the probationers sent by the institute have received in Brighton. In fact, it is clear that the shamefully inadequate support given to the Association can be explained on no other ground than that of indifference or selfishness. Mrs. Goslett, who also spoke at the meeting, said she was shocked at its financial condition "in a wealthy town like Brighton"; and making full allowance for the number of empty houses, it is obvious that the rich and well-to-do people who certainly reside there could, without any sacrifice, remedy a condition of things which, as the treasurer very properly says, cannot go on. If the subscription list be not largely and speedily augmented, some of the sick poor will have to be deprived of the advantages of being nursed in their own homes.

### NOT WANTED IN SOUTH AFRICA.

On her arrival at Capetown, Miss Hobhouse stated that her object in coming to South Africa was "to nurse the sick of the British refugees." The sick refugees, whether British or Boers, are provided by the British Government with trained nurses, whose duties, judging by the contents of the "Blue Book on the Refugee Camps," are often by no means light or pleasant. In view of the enormous sum spent upon the wives and families of men who are fighting against us, it is scandalous that Dr. Kendal Franks should have had to report to the authorities that the staff of nursing sisters at the Middleburg camp had no decent accommodation. However, according to the general superintendent of refugee camps, the improvements suggested by Dr. Franks will be carried out "as far as possible." The British public, who pay the bill, will be extremely indignant if, while their money is being poured out like water to make Boer women and children more comfortable than they have ever been in their lives, British nursing sisters are denied even the ordinary necessaries of civilised existence. Certainly, the sisters who are serving in South Africa will not have to complain of lack of variety in their work. In addition to nursing sick and wounded soldiers, and teaching Boer women how to be clean, some of them have had to be responsible for the safe detention on board the hospital ship of the enthusiastic lady who, with ill-directed zeal, wished to share their duties.

### A START AT RUABON.

The movement in favour of the establishment of a nursing association for Ruabon has made such good progress that at a meeting the other day it was unanimously resolved to procure the services of a trained nurse for the district. Sir Watkin Williams Wynn and other landowners have promised assistance, the Wrexham Board of Guardians will contribute, the Ruabon School Board have offered help from the proceeds of the dancing classes, and a recent cycle carnival yielded the sum of £17 10s. With £60 in hand, the strong committee which has been formed are fully justified in engaging a nurse.

### Lectures to Nurses on Medicine and Medical Nursing.

By NORMAN DALTON, M.D. London, F.R.C.P., Physician to King's College Hospital.

### LECTURE XXIV.—CONVULSIONS—EPILEPSY—HYSTERO-EPILEPSY.

EXCITATION of the motor functions of the brain and spinal cord gives rise to involuntary contractions of the muscles, which are called "spasms." These spasms are of two kinds. If the muscles remain rigidly contracted for some little time, so that the limbs or trunk or facial muscles are fixed in the position into which the muscular contraction has thrown them, the spasms are said to be tonic. Tonic spasms are most often met with in diseases such as tetanus (lock-jaw) and strychnia poisoning which affect the spinal cord and medulla oblongata. In lock-jaw, the muscles keep the jaw clenched continuously, and the mouth is drawn open into a perpetual grin, called the "risus sardonicus." Also the body may become arched, so as to rest only on the head and the heels, a position which is called "opisthotonos." Tonic spasms also occur at the outset of an epileptic fit and in hystero-epilepsy, which are brain diseases or perhaps diseases of the medulla oblongata. On the other hand, if the muscular contractions and relaxations alternate with great rapidity, so that the limbs are rapidly jerked about and the features twitch, the spasms are said to be "clonic." Such clonic spasms are common in brain diseases, and constitute what are called "convulsions or fits." The causes of convulsions are: (1) Certain organic diseases of the brain; (2) poisons in the blood which affect the brain; (3) irritation in some part of the body which acts on the muscles reflexly through the brain.

The organic "cerebral diseases" which cause convulsions are those which irritate the brain substance, such as tumours, abscesses, meningitis, etc. But even these lesions, as a rule, only produce convulsions when they are situated in certain parts of the brain, namely, the portion of the surface of the brain around the fissure of Roland (at which place the motor impulses are originated) and the pons. In meningitis the whole surface of the brain is irritated. The "poisons" which cause convulsions are those which exist in the blood in uræmia, and, sometimes, in other diseases. For instance, the convulsions which may occur in children at the beginning of measles, scarlet fever, etc., are probably due to the first rush of the poison of the disease through the capillary blood vessels of the brain. This in adults only causes a rigor, but in children the effect is so great that a convulsion results. It has been shown by experiments on animals that, if the brain be deprived of blood altogether by tying the arteries which supply it, convulsions result and it is believed that anæmia of the brain is, at least, a predisposing cause of convulsions. The "irritations which cause convulsions reflexly" are those which occur in the gums during teething, and, more rarely, in the skin and viscera from certain unknown causes.

Epilepsy stands by itself as a disease in which convulsions occur from time to time. We do not know the cause of epilepsy, and we cannot, therefore, place the disease under any of the three headings mentioned above. We know, however, that there is no organic lesion of the brain to account for the fits, and, also, that at the beginning of the fit the brain becomes anæmic. In a typical epileptic fit the patient may have some warning, such as a tingling or numbness of a finger, or a peculiar sensation about the throat, etc., which tells him that the fit is coming on. This is called the "epileptic aura." But it only precedes the fit by a few seconds. Whether there be an aura or not, as soon as the fit comes on the patient becomes unconscious, and, if standing, falls down. He may emit a cry. All his muscles become tonically contracted, so that the body and limbs are fixed in some contorted position. As the respiratory muscles are fixed, the patient cannot breathe and his face becomes purple, and were it not that this stage of the fit only lasts for a few seconds he would die. This, the first stage of the fit, is called the "tonic" stage, and it soon passes into the second or "clonic" stage, during which the face exhibits the most horrible grimaces and the limbs are jerked about, sometimes so violently that dislocation of the joints takes place. The tongue may be bitten in this stage or, at the beginning, when the jaws are first clenched. The second stage may last a few minutes; then the movements cease, and the patient, as a rule, sleeps heavily for some hours. It must be remembered that the above described epileptic fit has become the type of convulsions. Epilepsy is a disease in which these attacks occur at variable intervals without, as I have said, there being any lesion of the brain, any poison (so far as we know), in the blood, and without any irritation acting reflexly. When, therefore, convulsions of this kind occur in a disease (such as a cerebral tumour, uræmia, or inflammation of the gums from teething), which we know is not epilepsy, we call them "epileptiform convulsions," and to avoid confusion the word "eclampsia" has been introduced as a synonym for epileptiform convulsions which are not due to epilepsy. In severe cases of hysteria, convulsive seizures occur which are so like epilepsy, that the affection is called "hystero-epilepsy;" and if a nurse be the only witness, she may, by careful observation and accurate description, enable the doctor to say whether the case be one of true epilepsy or of hystero-epilepsy. It may be a very difficult matter to decide, but it may be stated that unless the fit presents the regular features of epilepsy—which are complete unconsciousness, a short tonic stage with blueness of the face, and a longer, clonic stage—it is always permissible to doubt whether the case is one of true epilepsy. In hystero-epilepsy the regular sequence of the stages is not observed and there is far more tonic spasm, so that the limbs and trunk are kept rigid and contorted, not merely at first, but at successive intervals during the fit, and blueness of the face from asphyxia does not occur. Also, when the muscular contractions are not tonic they frequently are "purposeful," that is, the patient may clasp the bedrails or anything within reach, may pinch or bite her flesh, or tear out her hair, none of which things are done in true epilepsy. It is more difficult to be certain about the state of the mind. In true epilepsy there is complete unconsciousness, and the old test of putting salt in the mouth evokes no response. Now in hystero-epilepsy the salt test, or douching with cold water, or applying the electric battery may, in slight cases, elicit some response from the patient, but they may fail in bad cases. In bad cases, too, the sensation is so blunted that a needle or knife may be passed through the fleshy part of a limb without the patient feeling it and without any flow of blood. Professor Charcot taught that pressure on the ovarian region in the lower abdomen would bring on, and would also stop, a hystero-epileptic seizure. I have known it to do the first, but have never known it bring the fit to a close. The hystero-epileptic seizure may last for hours, whereas a true epileptic attack lasts as a rule only a few minutes. However, occasionally, one true epileptic fit is succeeded rapidly by others, a very grave condition, which is usually fatal, and is called the "status epilepticus." Charcot states that the status epilepticus can be distinguished from the prolonged hystero-epileptic seizure by the fact that in the former the temperature invariably rises. So nurses should always take the temperature in these cases, if they can possibly do so. Associated with hystero-epilepsy are such well-known symptoms as the "globus hystericus," that is a sensation of a ball in the throat, which can neither be swallowed or brought up; the "clavus hystericus," that is a sensation of a nail being driven into the forehead, etc.

Nothing can be done for a patient during convulsions, whether epileptic or not, except to place the patient in a position where he cannot injure himself and cannot fall. It is usual to put some soft tough substance, such as a rolled bandage, between the teeth to prevent the biting of the tongue. It is also usual to place a child, when in convulsions, in a warm bath.

### Midwifery Unusual!

By a Correspondent.

We all know the work of the ordinary midwife, but just occasionally one is suddenly waked up by the consciousness that the work of the midwife may be quite different in different communities.

I am not speaking of barbarous countries, but of those where a high state of civilisation prevails, and where in the majority of things English methods are taken as the basis to work upon.

I have just had one of the doctors in Bhavnagar calling on me, and as we sat on the verandah looking out upon the fields of bari and jowarri and maize, and the gardens of limes and custard apples and capsicum and sugar-cane, we got into a discussion on the midwifery nursing of the city.

It began in a way quite incidentally, for he had been saying that the native charge nurse at the hospital received a salary of a pound a month, and an allowance of another pound a month for carriage expenses—being non-resident and having to find her own board and lodging—and I had musingly rejoined :

"Only £24 a year, without uniform, laundry, board, or residence," when he added, with a little air of apologetic triumph :

"Ah, but our midwife gets £2 13s. 4d. per month."

"Oh, midwife?" I asked eagerly. "I hadn't heard of this branch of your very meagre nursing as yet. Pray tell me who she is, and what she does."

"She comes to the hospital from 8 A.M. to about 9.30."

"To the hospital? But yours is not an obstetric hospital.'

"Oh no, she comes to examine female patients. You see our poor out-patients come from all castes. Well, women of the low castes don't mind being examined by a man, but to a high-caste woman the idea is so repugnant that if it were compulsory she would not attend the hospital at all. Of course, if the man were a Brahmin it would not be so bad, but for a woman, to whom all these things are abominations from birth, to be examined by a man who may smell of spirits, or reek of tobacco, or emit the odour peculiar to eaters of flesh meat, is too dreadful. These women are very sensitive about these matters."

"Well, what happens? I don't see quite what you mean. In England, too. there is always a nurse present when a woman is being examined."

"Yes; but here the midwife examines the patient and then comes and reports to the doctor and he prescribes."

"How was your midwife trained for this work?"

"Oh, she was trained at the Jamsetjee Jheejeebhoy Hospital at Bombay; and, of course, after a long practical experience, she can generally diagnose the usual vulvular and vaginal troubles."

"Clever woman," I thought to myself, as I mentally ran over the puzzling cases one is constantly getting in both hospital and private practice. "And after 9.30 A.M. what does she do?"

"She goes to the State Dispensary, and does the same work there up to about 10.30, and then, if necessary, comes back here again for half an hour or so in the evening."

"But why *midwife*?"

"Because, in addition to this work, for which she is paid as a sort of retaining fee, she has to attend all confinements to which she may be called."

"Does she charge for this?"

"Yes; half-a-crown for each delivery."

"Twins five shillings then, I suppose?" But he was not a humorous man and did not see the joke.

"Have you no free midwife for the poor then?"

"No. Half-a-crown is the fixed charge, and the poor do not usually have a midwife."

"Nor a doctor?"

"No, certainly not. They are more often than not delivered in the fields, and after a little rest the woman goes on with her work. You must remember," he went on, "parturition is not with our poor the dreadful thing it is with you. To them it comes all in the day's work."

"Why do you 'charge half-a-crown for delivery?' I suppose the midwife attends each case for several days after the actual delivery?"

"Not unless the people pay extra. The half-crown is the usual fee, and then the midwife's duty entirely ends. If, however, they wish her specially to call again they pay an extra 8d. per visit."

This is the first step made by the State to introduce trained obstetric nursing. Up to now there were only the ordinary "neighbours" type of nurses, and it says much for the parturient capacity of the people that the mortality of childbirth has been so low. Mortality after childbirth has been much higher because surgical cleanliness has not been so carefully practised, but amongst the higher classes this has become well understood, and ere long puerperal fever should be as rare in India as it is in similar ranks of life in England. Forty thousand inhabitants and one trained midwife, and she needing a State retaining fee! It is a beginning.

### Examination Questions for Nurses.

### THE FEEDING OF TYPHOID FEVER PATIENTS.

The question was as follows: "Supposing yourself called to nurse a case of severe typhoid fever without medical direction, how would you feed the patient, whilst liquid nourishment alone was permissible?"

No Prizes Awarded this Month.

No answers sent in merit a prize. Taken as a whole they are disappointing. Consider the question carefully; it says you are to feed your patient "without medical direction." In outlying districts and also when an epidemic is severe, the doctors have little time to enter into minute particulars concerning diet, and very generally leave the matter to be settled by the nurse, who is supposed to be capable of undertaking such responsibility. No one seems able to go beyond the stereotyped three or four pints of milk diluted with barley water, etc., . . . and the pint of beef tea or chicken broth. One bold spirit advocated iced champagne, which was a move in the right direction. When you are tied to an absolutely liquid diet, but are not hampered by directions, there are at least forty or fifty different things that can be given, some of them extremely nourishing, whilst others are only stimulating or simply useful as allaying thirst. If I tell you these things the next answers sent in to the repeated question will be mine not yours. Study the matter well and I hope that in the Spring, when the question will be given again in the same words, the result will be very much better. Patients fed as most of you suggest would be rebellious and sulky before three weeks were out. All give the regulation diet—and quite right too—but there are harmless and permissible varieties of this which it is essential that every nurse should become acquainted with.

HONOURABLE MENTION.

"Devonia," "Nurse Gertrude," "Manningham," and "Ecila" have all gained honourable mention. "Devonia's" answer is the best, she ventures off the eternal track of milk and broth. "Nurse Margaret" sends a good answer, but written in a form too voluminous for printing; such minute particulars concerning quantities are unnecessary. The same may be said of "F. H. D.'s" paper.

OBSERVE THE RULES.

I have received 39 requests for rules; these cannot be sent privately. They were unfortunately omitted on October 5th, but it surely would have been very easy to look at a back number of the paper. Do not send in papers without putting "Examination" in the left-hand corner of the envelope, nor write letters with your answer. If your name and address is not put on the answer itself, that answer goes immediately into the waste-paper basket.

HOW TO MAKE MILK TEA.

One nurse speaks of this agreeable change in the way of giving milk, but she makes it quite wrongly. Swell the tea first by putting about an ounce of boiling water on the leaves, let it stand three minutes, and then add the hot milk. For some chemical reason boiling milk does not uncurl or soften the leaves, and without using water first, you do not get tea at all, but simply dirty-looking milk with tea stalks floating on the top.

QUESTION FOR NOVEMBER.

1. What symptoms would cause you to suspect a case of small-pox? 2. State the difference between a slight and a severe case. 3. What are the chief difficulties to be encountered in the nursing? How would you feed the patient? In all questions relating to diet it must be understood that where a medical man gives orders, those orders must be carried out with the utmost exactitude, no deviation from the prescribed quantity or quality being attempted, but very often (especially when a serious epidemic is present) the doctor has but little time, and leaves the diet question to his nurses, and then thoughtful intelligence is wanted.

UNAVOIDABLE WANT OF EXPERIENCE.

Small-pox has fortunately become a rare disease, and few nurses have had opportunity of personally observing it; it is therefore impossible to the majority to answer this question without resorting to the help of books, and though that is a course strongly deprecated generally, the matter is of such vital interest just now that I feel it should be dealt with.

THE EXAMINER.

RULES.

The competition is open to all. Answers must not exceed 500 words, and be written on one side of the paper only. The pseudonym, as well as the proper name and address, must be written on the *same* paper, and *not on a separate sheet*. Papers may be sent in for fifteen days only from the day of the publication of the question. All illustrations strictly prohibited. Failure to comply with these rules will disqualify the candidate for competition. Prizes will be awarded for the two best answers. Papers to be sent to "The Editor," with "Examination" written on the left-hand corner of the envelope.

N.B.—The decision of the examiners is final, and no correspondence on the subject can be entertained.

In addition to two prizes honourable mention cards will be awarded to those who have sent in exceptionally good papers.

### Bananas and How to Use Them.

BY A CORRESPONDENT.

As bananas, a once rather rare, and always delicious, fruit are now coming to us in such abundance, it will not be amiss to consider how best it can be utilised for many classes of patients.

I have read of bananas being used most successfully in typhoid cases, but refrain from recommending them on mere hearsay, though we hear from South Africa of recoveries from enteric on foods that would be thought impossible in England. Before long I hope we shall have a plentiful supply of banana flour in the market, the sustaining powers of which we can read of in many books on travel.

I know from personal experience the hours of fatigue people can undergo, taking no other food but bananas. There is a great charm about freshly-cut fruit that has been sun-ripened, and not cut green and allowed to mellow and ripen by age. Now that ships fitted with refrigerators bring us fruit we shall have it in much greater perfection, and every year the supply is larger and better. In fact, we may look forward to the time when bananas will be cheap enough for all classes to buy. Every part of the fruit is valuable. The inner skin is an excellent preservative for undressed leather, and improves the look and wear of brown boots and shoes, which should be rubbed over with the soft part of the banana skin, then polished with an old silk handkerchief. The following recipes are very good and make a nice change from giving patients the fruit in its raw state.

BANANA JELLY.

Rub three ripe bananas through a wire sieve, after removing the skins; put the pulp into a basin, add to it half a pint of liquid lemon jelly, stir together till cool, and when beginning to set put the mixture into a small fancy mould; set aside till cold, then dip the mould in warm water, wipe it with a dry cloth, and turn out on a glass dish.

BANANA SOUFFLÉ.

Rub a sufficient number of ripe bananas through a wire sieve to make a quarter of a pint of pulp; put this in a stewpan with one ounce of flour, half an ounce of butter, two tablespoonfuls of cream, three ounces of castor sugar, two raw yolks of eggs; stir over the fire till the mixture thickens, then add two bananas sliced, and two whites of eggs that have been whipped stiff with a pinch of salt. Mix altogether gently, and pour into a buttered soufflé-tin or pie-dish, and bake like an ordinary soufflé for 20 minutes or half an hour.

BANANA CREAM.

Rub four bananas through a sieve, whip a quarter of a pint of thick cream till nearly stiff, stir in six sheets of leaf gelatine that has been melted in a little warm milk, sweeten it with a tablespoonful of castor sugar, adding a few drops of vanilla essence. Mix the prepared cream and banana pulp together, and put into a small fancy mould, or serve in glasses when cold.

BANANA COMPÔTE.

Slice two or more ripe bananas into a glass dish, sprinkle them with a little castor sugar, a few drops of lemon-juice and, if liked, a little cognac; stand aside in a cool place or on ice till wanted.

BANANA FRITTERS.

Make a frying batter by mixing one raw yolk of egg into an ounce of floor, add a little milk and the white of the egg whipped stiffly with a pinch of salt; remove the peel from two bananas, and slice the fruit straight down the centre; dip these in the batter and fry in clean-boiling fat till a nice light brown; serve with castor sugar sprinkled over the fritters.

BANANA MERINGUES.

Remove the centre from two penny sponge cakes, fill the centre up with a purée of banana, seasoned with sugar and a little lemon-juice. Whip a white of an egg stiffly with a pinch of salt, and mix lightly into it a tablespoonful of castor sugar. Cover the top of the purée with this meringue and place the cakes in the oven for a few minutes, it must be but moderately heated; serve either hot or cold.

### How to Use the Clinical Thermometer.

By Eldon Pratt, M.D.Lond., Honorary Anæsthetist, Cardiff Infirmary.

It is unfortunately too well known that the clinical thermometer gets broken far too often; this is frequently due to carelessness. While recently holding the position of resident medical officer in a large provincial hospital, it came to my notice that the number of thermometers broken was alarmingly great; an experience, to my knowledge, shared at many other hospitals. I began to think, therefore, how one could lessen this unnecessary expense; the following rules are the result of such thought. They have been in use now nearly two years, and have certainly had the desired effect of reducing very considerably the number of breakages—a saving both to the institution and to the nurses themselves. So useful have I found these rules that I thought they might be of benefit to others. In the institution where I brought them out they hang framed in each ward, and every new probationer receives a copy of them from the matron on the commencement of her duties.

1. Remember that a clinical thermometer is a thin glass instrument and is very liable to be broken if handled carelessly. It may last for years if properly used.

2. Of clinical thermometers we have two kinds, according to the rapidity with which the record of the body-temperature is obtained. (*a*) The "half-minute" thermometer registers the body-temperature in 30 seconds, and is an extremely delicate instrument, and is more than twice as costly as the ordinary thermometer. (*b*) The "ordinary" thermometer is a less delicate instrument, but nevertheless requires very careful handling; it takes at least 5 minutes to give the body-temperature.

3. The body-temperature may be taken in several places, especially in the axilla, under the tongue, and in the rectum. It is in the rectum that the true temperature of the body is recorded, but for convenience it is usually taken either in the axilla or under the tongue.

4. When taking the temperature in the axilla the best position in which the thermometer should lie is with the bulb high up in the axilla and the stem lying against the side of the chest. It will then be observed that when the arm is brought to the side, the body, the thermometer, and the shaft of the humerus all lie parallel, and so long as the elbow is held firmly against the side no harm can result to the instrument. At the end of half a minute, or 5 or 6 minutes, as the case may be, the thermometer is removed and the result noted on paper at once. The mercury is then shaken down as low as it will go, and the instrument cleaned and replaced in a proper receptacle.

5. If the temperature be taken under the tongue, it is better for the patient to be lying down, or at least he must be in a position of rest. The bulb of the thermometer is then placed under the tongue, the stem being brought out at the angle of the mouth, and the lips are then firmly closed. The patient must be warned not to bite the instrument. After noting the temperature the same precautions of shaking, cleaning, and replacing the instrument are performed as mentioned above.

In certain cases it is necessary to take the temperature in the rectum, when similar precautions are adopted.

6. If a "half-minute" thermometer is used, and this is advised, it is necessary for the nurse to remain by the patient, watching him, until the temperature has been taken. If an ordinary thermometer were used, this would take too much time, hence the advisability of using the more expensive instrument, which thereby saves time and is probably also more accurate; but it must be left in a full half-minute.

7. Having removed the thermometer, do not make up the patient's bed with the instrument in your hand or between your teeth;* such habit is dangerous as well as unclean, since it might so easily get broken. Always at once replace it in its proper place after shaking and cleaning.

8. Unless you use a proper case, the receptacle for thermometer should be a small glass bowl or tumbler with cotton wool at the bottom, and the vessel half-filled with 1 in 20 carbolic acid lotion. Wipe off the lotion before using the instrument. In a ward a small bowl is always used.

9. Follow these directions and your thermometer will last for years.

10. It is the duty of the sister of the ward to teach each fresh probationer how to use the thermometer. The nurse using the thermometer will be held responsible for the same so long as it is in her care.

* I have actually known this done.

### Appointments.

Aston Union Workhouse Infirmary.—Miss Grace Kirby has been appointed charge nurse. She was trained at Southwark Infirmary, East Dulwich.

Cheltenham Workhouse Infirmary.—Miss M. E. Oakey has been appointed superintendent nurse. She was trained at Worcester General Infirmary, where she was afterwards staff nurse. She has since been sister at Birmingham Infirmary.

Hanwell Cottage Hospital.—Miss Helen C. Vokins has been appointed matron. She was trained at Birkenhead Borough Hospital and Queen Charlotte's Hospital, London, and has since been assistant nurse at Dorset County Hospital.

Hemel Hempstead Workhouse Infirmary.—Miss Mary R. Skinner has been appointed nurse. She was trained at the Mildmay Institute and the Cancer Hospital, Brompton. She has since been nurse at Scarborough General Hospital, and attached to the White Cross Nursing Home, Norwood, and Baker Street Nursing Association.

Stirling Royal Infirmary.—Miss Jane Peebles has been appointed matron. She was trained at the Royal Infirmary, Dundee, where she has held for the last eight years the post of sister of the accident ward.

Worcester General Infirmary.—Miss Daisy Garstang has been appointed sister. She was trained at the Blackburn and East Lancashire Infirmary. She has since been charge nurse at the South-Eastern Fever Hospital, London, and has done private nursing for eighteen months.

Yorkshire Cottage Hospital, Bridlington.—Miss Elizabeth Holliday has been appointed matron. She was trained at the General Infirmary, Bradford, and has since, for nine years, been night superintendent at the Bristol Royal Hospital for Sick Children and Women, during two of which she acted as assistant matron.

### To Nurses.

We invite contributions from any of our readers, and shall be glad to pay for "Notes on News from the Nursing World," or for articles describing nursing experiences, or dealing with any nursing question from an original point of view. The minimum payment for contributions is 5s., but we welcome interesting contributions of a column, or a page, in length. It may be added that notices of entertainments, presentations, and deaths are not paid for, but, of course, we are always glad to receive them. All rejected manuscripts are returned in due course, and all payments for manuscripts used are made as early as possible after the beginning of each quarter.

### American Red Cross Sisters.

By a Correspondent.

The Red Cross Hospital in New York City gives a special course of training to fit nurses for duties connected with war, pestilence, famine, or any other national calamity. Comparisons are said to be odious, and "Little Englander" is the first accusation awaiting anybody who extols the virtues of other countries. On the contrary, to wish one's own nation to excel in all points is to be the biggest of "big Englanders," and Great Britain assuredly should possess a standing organisation of nurses—similar to that of the American Red Cross Sisterhood—whose services could be commandeered for local epidemics, pestilences, and all occasions calling for the assistance of certificated nurses. Our Army Reserves even were but scantily recruited until war was upon us. I was engaged throughont the great Maidstone enteric outbreak, and I could not help feeling that some organised society should be called into existence to whom application might be made for nurses trained and competent to face such a grim situation. I was also in Hamburg during the historic and unprecedented cholera epidemic of 1892. Stray volunteer nurses from foreign countries and scattered training schools afforded their unpaid services to the cholera stricken city. Helpers — male and female, trained and untrained—were gathered in from all quarters. There was no plan, system, nor nursing co-operation. The lack of a civic or national nursing organisation was painfully apparent. I have also been many times on the spot at similar crises of a local or national character in the United States, and have been struck by the ease with which it is possible there to draft a body of well-trained nurses *en masse* to the scene of a disastrous flood, a big railway smash-up, or a wide-spread epidemic.

A Condition of Service,

All the nurses enrolled as American Red Cross Sisters must hold themselves ready to come up when called upon to serve their sick and wounded countrymen at home or abroad. The sisters receive no salary, but whilst on active service they are boarded, lodged, and provided for. They are pledged to face uncomplainingly all privations, dangers, and hardships encountered in the course of their duty. It is enough that their President "the Right Honourable" Clara Barton issues an order. It is their province to obey. Nurses who are specially interested in this form of public work can take their entire training at the Red Cross Hospital in New York City. The course extends over some two years and three months, and is adapted to the special needs of military and public service. Ambulance work, first aid, stretcher-bearing, and field and camp hospital methods are fully taught. Graduates from other recognised training schools are also admitted to Red Cross ranks, but only on the satisfactory completion of a six months' special course at the Red Cross Hospital.

The Uniform.

When the sisters "go to the field," and this may mean being drafted off to help a neighbouring city after a big fire or during a serious epidemic, or it may entail a term of service in Santiago, the becoming Red Cross uniform is worn. It consists of a navy blue gown, white belt, large linen apron, and the snowy kerchief so beloved of American nurses picturesquely crossed on the breast. One of these kerchiefs folded into a mitre shape, somewhat after the fashion of a serviette on a dinner-table, forms a most attractive cap.

The Record.

The society has been in existence some eighteen years and its nurses have performed much splendid work in that space of time. They have carried nursing relief, food, and clothes to sufferers from the devastating forest fires so common in the States. During cyclones, floods, and earthquakes they have taken express trains across the vast continent to give their skilled services to the sick, injured, and homeless. They have fed the hungry with good things, and restored the famishing with tender care and suitable diet in the Texas famine of 1885. Miss Barton took a corps of her Red Cross sisters to help the Russians in their long famine which began in 1891. She and her faithful followers did sturdy service among the persecuted Armenians, and saved hundreds of lives during the recent starvation and pestilence in Cuba.

A Stringent Rule.

In times of peace anybody who so chooses may adopt the uniform of the society just as any American citizen may deck himself out, an' it so please him, in the uniform of a field marshal. But the wearing during a war of an unauthorised Red Cross garb or military uniform is an indictable offence. A stringent rule of the society is to the effect that any sister on active service who attends first to a sick or wounded countryman of her own instead of to the person lying nearest to her is immediately called upon to resign. Candidates for the Red Cross sisterhood are not asked their religion or nationality. All classes and creeds are welcomed. The unfit are weeded out during their course of training. Preference is invariably given to nurses accustomed to assisting at major operations and laparotomies, and familiar with the routine of abdominal surgery which plays so leading a part in field and base hospitals. Promotion is gained partly through passing examinations and partly by distinction during actual service. There are four rank grades ranging downwards from the sister-in-chief, sister, to associate sister. It is an admirable association, and I should like to see a similar Imperial Red Cross Sisterhood formed which would include Great Britain and all our loyal colonies, with a specially drawn up course of training to fit its members for all the exigencies connected with war-making by land and by sea, and for every possible form of national disaster.

### Presentations.

Memorial Hospital, Kendal.—Miss Evelyn Hurlbatt, on her retirement from the post of matron Memorial Hospital, Kendal, after nearly four years' work, received a leather case containing 23 sovereigns from the ladies' committee, a clock in silver-mounted case from nursing staff, a silver card-case from a member of the medical staff, a travelling-rug from the hon. secs., and many other personal gifts, including a gold curb bracelet, and a turquoise pendant.

### Death in Our Ranks.

We regret to hear of the death of Miss Elizabeth Campbell Darrock, who was attached to the staff of the Manchester and Salford Sick Poor and Private Nursing Institution. Miss Darrock was trained at the Pendlebury Hospital, and her death, which occurred on November 14th, was due to enteric fever, contracted whilst she was nursing cases at the Scarborough Sanatorium. The funeral took place at Scarborough last Friday, in the presence of members of her family and a number of Manchester and Scarborough friends, all of whom contributed wreaths.

### Nurses in Public Schools.

By a School Matron.

There can be no doubt that the health of the boys in our great public schools receives far more attention now than it did fifty or even twenty years ago. There is a growing inclination on the part of the masters to engage as matrons only such persons as have had a certain amount of hospital training, and it is no uncommon thing for a girl who has been, say, a governess and who wishes to take up the work of a school matron, to go first to some provincial hospital for three months in order to qualify herself for the post. This is quite as it should be, and no doubt the health of the boys benefits greatly by the change from the "Gamp" of the past to the trained, or even partly trained, nurse of the present. The work of a school matron is of course only in part nursing, and sometimes she may have little or no sickness amongst her boys; but what she chiefly has to do is to keep an eye on them when in health, to decide whether a boy with a headache or a gathered finger is well enough to go into school, and above all to detect the first suspicious symptoms of any infectious malady. In most schools there is a general sanatorium, with a fully-trained nurse in charge, to which boys who are at all seriously ill are sent. Should an epidemic break out, additional nurses are engaged from some private nursing institution. The post of sanatorium nurse in a large school is undoubtedly a very interesting one, and is well suited to a nurse no longer very young, who perhaps cannot stand the perpetual strain of hospital life or the bad weather of district life, or the constant changes of private nursing. Such a nurse should of course be fond of boys and should have some hobbies or interests of her own to occupy her spare time, as there may be long periods of inactivity in the sanatorium. The salaries of these posts are generally good—from £50 upwards—with comfortable, if somewhat isolated, surroundings.

### The Work.

But it is less of the sanatorium life that I wish to write than of the work of a school matron, in dealing with the important question of her boys' health. In a school such as I have in view a great deal depends on the matron. She is probably the only gentlewoman with whom the boys come into contact during their school life—at any rate if the house master is unmarried. To her come the home-sick little new boys for a cosy talk; to her also the exalted sixth form boys to discuss subjects other than football. At her tea table manners are remembered, and in her drawing-room spotless collars are essential. Anxious parents write to her, the servants appeal to her, and probably never a day passes without her having to treat, to the best of her ability, the minor medical and surgical ailments of her numerous family. Questions of diet, ventilation, sanitation, etc., are also largely in her hands, and if she is a trained nurse, there is very little of her training and experience that is not in request at some time or another. A knowledge of human nature, and especially of boy nature, is a necessary qualification, too, if she is to give a just answer to a boy who asks to be excused his work. Some boys will try to evade both work and games, and will come with solemn faces, say that they feel "a little sick," or "rather bilious," and ask for an excuse. Others will finish their lessons at all costs, in spite of a splitting headache, and will throw themselves into football, notwithstanding a sore throat and a heavy cold. The causes of their ailments, too, are often obscure, but it is most necessary to know them. A certain boy came to me the other day with a sad story of severe sickness and diarrhœa. Of course bed was the only possible remedy, and work was knocked off. On inquiry I discovered that he had just had a birthday, and that a fond mother had sent him a hamper of delicacies. *Apropos* of this, a remark by another boy was overheard in the kitchen regions, and passed on to me. "It's no good our shamming, she understands us too well, and we only get starvation!" Trifling as the indisposition of boys often is, there is the continual possibility that it may be the beginning of something serious, and great vigilance is necessary in order not to overlook important symptoms. Since I have been here, I have discovered that it is quite a common thing for a healthy boy to have a sore throat, headache, and temperature of 100 or 101, which develop into nothing at all. But just now and then it happens to be measles or scarlet fever, and then one is glad to have taken every precaution, so that no second case occurs.

### The Edict of Revaccination.

Some variety to the usual routine was caused not long ago by an edict going forth that everyone should be re-vaccinated. Then ensued quite a busy time and batches of bare-armed boys were waiting about every evening for their turn to be "done." Care was needed here too, for an astonishing number of strong boys faint during or after the process. Whether this is due to "nerves" or not I cannot say. There is indeed no knowing how much or how little it takes to make a boy faint. A local doctor told me not long ago that he has twice seen boys faint on having a thermometer put into the mouth!

### Surgical Cases.

There are a certain number of surgical cases, but on the whole not so many or so serious as I had expected. Whether the human boy is more tough or more careful than he used to be I cannot determine, but certainly I am surprised to find how seldom he gets damaged. Not a single fracture has occurred in my time except a nose, and that is such a poor sort of fracture to tend! There was one rather serious cut in the summer time—a boy bathing trod on broken glass and had some fairly copious hæmorrhage. Another boy crushed his hand badly through accidentally letting a heavy weight fall on it, but football, which looks so murderous, does not seem to inflict any damage, and though I am constantly expecting to see a stretcher brought in, such an event has never yet happened. To a fully-trained nurse well up in her work and accustomed to a life of excitement and activity, such a post as that of a school matron might seem a little tame and dull. But on the other hand, to one who is fond of boys, who welcomes periods of quiet, and who cares for the larger aspect of the influence of women on boyhood, the life is full of interest and possibilities, while the prevention of illness by the careful consideration of hygiene, and the ready application of knowledge in cases of emergency, is perhaps of just as much importance as the actual nursing of patients seriously ill.

### Where to Go.

Empress Rooms, Royal Palace Hotel, Kensington, Wednesday, December 11th. Ball in aid of the funds of the Royal London Ophthalmic Hospital, City Road. Dancing at 10 p.m.

New Gallery, 121 Regent Street.—Society of Portrait Painters, Autumn Exhibition, 10 to 6.

Town Hall, Stratford, Monday and Tuesday, November 25th and 26th. Bazaar in aid of the Maternity Charity and District Nurses' Home, Howard's Road, Plaistow.

### Everybody's Opinion.

[Correspondence on all subjects is invited, but we cannot in any way be responsible for the opinions expressed by our correspondents. No communication can be entertained if the name and address of the correspondent are not given, as a guarantee of good faith but not necessarily for publication. All correspondents should write on one side of the paper only.]

### THE NURSE'S NEVER.

"G. M." writes:—"Brighouse" has evidently not had my experience as to water bottles. Within twelve months I have had two cases of patients who were badly injured by insufficient precautions being taken by trained nurses in putting water bottles in beds. In one instance the patient speedily recovered from the surgeon's operation, but had to remain in bed some weeks in order to recover from the scald caused by the water bottle; and in another—a medical case—a similar thing happened. The nurses had been thoroughly trained in different hospitals, each could show splendid credentials, but somehow the mistake, equally deplored by patient and nurse, occurred.

"Nurse Prudence" writes: Having had an experience of nearly 14 years' hospital and private nursing, I should like to know to what class of nurse "The Nurse's Never" is addressed, as I have never heard that it was considered necessary to take the temperature of a dead patient. I should feel obliged if "The Medical Lecturer" would kindly explain "Never No. 5" of last week's lecture. Surely there can be no nurse who requires to be told "Never to allow a patient to be waked out of his first sleep, either intentionally or accidentally"; and again, "Never administer a quantity of food to a patient until you have found out if he can swallow. I think, too, that every nurse would measure "drops," when ordered, in a graduated measure, as well as the doses; and, with the exception of effervescing medicines, I never pour them out near the patient (that is a private patient), as I consider it has a tendency to nauseate them. I, with many other nurses, much regret that "Medical Lecturer" has concluded his series of lectures, as they have certainly been amusing, if not altogether instructive.

"Two Private Nurses" write: We quite agree with "A. R." that "The Nurse's Never" cannot be intended as suitable advice to probationers or the hospital staff; but neither, we think, can it be applicable or useful to the private nurse, whom we take it for granted has "passed the stage of being nursed intellectually and is qualified for her post," ere she is engaged as a private nurse. Our "Medical Lecturer" seems to have a very poor opinion of nurses in general, and appears to think that they are being or have been most inefficiently trained, and are quite devoid of even an ordinary amount of intelligence. As hospital training tends to sharpen the wits and develop the common sense of the average woman, a trained nurse generally has the advantage over the rest of her sex in intelligence and practical ability, though, we fear, our friend the "Medical Lecturer" does not think so. Surely the nurse who needs to be told to clean her own teeth, and not to use her patient as a "clothes horse," or to neglect washing him regularly, "is a rare and ridiculous sight." Can "The Medical Lecturer on Nursing" simply be perpetrating a huge joke?

### WHY ARE NIGHT NURSES NEGLECTED?

"Matron" writes:—"A Night Nurse" asks: Do matrons forget that they have been on night duty themselves? By no means. Nor do they forget that they, who were the pioneers of trained nursing, did indeed "endure hardness." Plain living and hard work is what they expected as hospital nurses, and they got what they expected. But where was the grumbling, where the complaints, where the grievances with which one is pestered in these days? Speaking for myself, I can truthfully say I do not remember ever hearing any. Why is this? Are nurses being spoilt? Are they deteriorating? These are questions we should ask ourselves very seriously. Why does a night nurse consider herself martyrised and unable to sleep because she has no bedroom fire? Does she get one at her own home? It seems that as soon as some women put on cap and apron they set to work to see how many causes for complaint they can discover. Nursing has become an exact science; let nurses beware lest by their want of sense they lose the respect which used to be theirs.

### THE WORKHOUSE NURSE.

"Justice" writes: I think it is quite time that something should be done to protect the workhouse nurse from the petty tyranny of the usual run of masters and matrons. It is rather hard, that a nurse, who has gone through her training, should have to be subservient to such people as these often are. In one of the unions where they keep a superintendent nurse as well as assistants, a clean dress cannot be provided for one of the patients, however necessary, without asking the matron, and it just depends on her opinion whether the patient gets it or not. Surely, where there is a superintendent nurse, she might be allowed a stock of everything for the infirmary, to be given out at her discretion. There will always be friction between nurses and untrained matrons if these matters are not remedied. Of course, if the matron were trained, she would know much better, and understand what was requisite. Until that time comes, I fear we shall never know what it is to have peace among the staff.

### CHRISTMAS PRESENTS.

"Misunderstood" writes: Now, when Christmas is drawing near, may I be allowed to write on a subject in which nurses everywhere are interested? I mean the giving and receiving of presents. After many years spent in hospitals I have found that the old and pleasant habit has degenerated into what may be looked upon as an almost compulsory custom. For nearly all nurses, and especially for the sisters of wards, it is nothing but a tax. Many nurses are quite unable to afford more than a small gift to their own particular friends, yet they find themselves compelled to subscribe largely and to give presents to matron, sisters, fellow probationers, and servants, who are in some instances perfect strangers, for the sake of "what will be said" if they abstain. Sisters particularly find themselves bound to give to each probationer, the wardmaids, and other servants, and in addition have calls on their purse for flowers, plants, and decorations for their wards. The custom is one which I believe none but the matrons themselves can put a stop to, and I do beg them in the name of my fellow nurses of all grades to at least try and minimise the amount of the tax. I would suggest that each matron should put up a notice in the nurses' dining or sitting room to the effect that she wishes in the future that only small presents to personal friends should be given at Christmas. I feel sure that many nurses of every staff would be most grateful to their chief for arranging a very difficult problem, viz., how to make £1 stretch out to £5.

### The Nurses' Bookshelf.

The Ideal Nurse. By Nurse Agnes. (Published by Nurse Agnes, Bourton-on-the-Hill, Moreton-in-the-Marsh, Gloucestershire. Price one shilling.)

Nurse Agnes's little book does not deal with the technical accomplishments of the "ideal nurse," but only with the spirit in which she ought to undertake her duties. While this little brochure gives no hints as to actual nursing, it is interesting as showing the sense of moral and religious responsibility which animates a pious and high-minded woman in relation to her work and her patients. A supplementary address to the "ideal doctor" demands of him the same regard for soul as well as body which is asked from the ideal nurse. When, however, Nurse Agnes says, "I do not think we do well to trust the lives of persons to any save thoroughly genuine Christian men," she raises a difficult problem, and one which she makes no attempt to solve. It is difficult for patients and their friends to judge of a doctor's professional skill, but it is infinitely more difficult—may we not say that to all but One it is nearly impossible ?—to judge of the genuineness of his Christianity, and such tests as may be applied are apt to result in the preferring of the unctuous charlatan to the man who does his duty in the matter of relieving pain and saving life to the best of his ability, without making any fuss about it.

The Commonwealth of Cells: Some Popular Essays on Human Physiology. By H. G. F. Spurrell, B.A.Oxon. (London: Baillière, Tindall and Cox, 8 Henrietta Street, Strand. 1901. Price 2s. 6d. net.

Mr. Spurrell's little book contains an admirable account of the human body, beginning with the protoplasmic cell, and going on to the more elaborate tissues into which these are formed. It is not technical; that is, no long words are used, and very few which are not comprehensible at the first glance to any person of ordinary education, while such special terms as cannot be omitted are clearly and simply explained. There are many diagrams, which are wisely diagrammatic, and do not give that "gruesome" feeling which the author has observed is aroused in the lay mind by a hasty glance into the ordinary text-books of physiology. None the less—perhaps all the more—do they make clear at a glance the great highways of life, the digestive, circulatory, and nervous systems. The essays on the chemistry and on the mechanics and physics of the body will help to make clear to many processes of life which are but vaguely understood. This is a very readable very interesting, very useful little book. Those who have had a training in physiology need not despise it, and those who have not, or who have had only a brief and sketchy course of study, should welcome it.

Advice to Women on the Care of the Health Before, During, and After Confinement. By Florence Stackpoole. Third Edition. Revised and greatly enlarged. (London: Cassell and Co. 1901. Price 2s.)

This is a new edition of a book which has had a certain vogue. It is not a very satisfactory performance. Beginning apparently with the object of teaching a woman how to lock after her health, it drifts round into a sort of handbook for the nurse, and then enters upon matters appertaining to the midwife. We think the book is too full of prescriptions. If it is a nurses' book they are out of place, if a mother's they are absurd. It is admitted that abscess of the breast requires a doctor; why then give prescriptions for tonics to be used in its treatment? Again, we fail to see the propriety of giving prescriptions for anodynes and sedatives for the treatment of after-pains. Such medical treatment is outside the province even of midwives; but here we are told that a teaspoonful of liquid extract of ergot may be used, or a pill containing a grain of opium, which "is perfectly safe and usually gives great relief," or a suppository of morphia, or a hypodermic injection of morphia "which gives sure and immediate relief, and is not attended with the least danger." Again and again this dabbling in physic is repeated. For example, a prescription is given for a "mixture for lessening discharge if too profuse," a mixture containing ergot and sulphuric acid, to be taken "three times a day until the coloured discharge lessens." Those who hold that the registration of midwives must act as a direct encouragement to unqualified medical practice will find in this book much to support their contention, for it appears that the authoress who recommends these things is a "Diplomée (sic) of the London Obstetrical Society." Some of the paragraphs dealing with disease are, to say the least, injudicious. Among the illnesses after confinement, "Weid or Ephemeral Fever (popularly called The Weed, or Milk Fever)" is described as having the following symptoms: "Rigor, headache, flying pains, fever, perhaps suppression of milk and lochia. There is a cold, hot, and sweating stage; during the profuse sweats of the latter the fever ceases. Much alarm may be caused by fear that puerperal fever is coming on, but the ague-like stages of 'The Weed' should dispel alarm." Should they? We doubt whether experienced accoucheurs would feel very happy with these symptoms before them, even though such evidences of septicæmia should show "ague-like stages."

### For Reading to the Sick.

### THE MASTER'S VOICE.

I listened there
With breathless longing by that solemn sea,
Till through the curtains of the night I heard
His own Voice calling me—that Voice which draws
His [illegible]ildren through the flood and through the fire
To ki[illegible]s His Feet; and at the Master's word
I left the shore, forth walking on the dim
And untried waters there to follow Him
Who called me, and there to see His Face. *B.M.*

---

In sickness I will be
Watching beside thy bed,
In sorrow thou shalt lean on Me
Thy aching head.
In every struggle thou shalt conqueror prove,
Nor death itself shall sever from My love.

O grace beyond compare!
O love most high and pure!
Saviour, begin, no longer spare!
I can endure:
Only vouchsafe Thy grace, that I may live
Until Thy glory, who canst so forgive.

*C. M. Noel.*

---

There is nothing that so brings near and invites the Man of Sorrows as grief; for He is Himself the great Comforter. No walk so ordinary, no business so constraining, but that as we walk the Divine Stranger may be with us, relieving our sadnesses, condescending to our ignorances, and sympathising with our weaknesses.—*Isaac Williams.*

---

The crosses which we make for ourselves by a restless anxiety as to the future, are not crosses which come from God. We show want of faith in Him by our false wisdom, wishing to forestall His arrangements, and struggling to supplement His Providence by our own providence. The future is not yet ours; perhaps it never will be. If it comes, it may come wholly different from what we have foreseen. Let us shut our eyes, then, to that which God hides from us, and keeps in reserve in the treasures of His deep counsels. Let us worship without seeing; let us be silent; let us abide in peace.—*Fenelon.*

---

There is in man a higher than love of happiness: he can do without happiness, and instead thereof find blessedness!

*T. Carlyle.*

---

The elements of *happiness* in this present life no man can command, even if he could command himself, for they depend on the action of many wills, on the purity of many hearts, and by the highest law of God the holiest must ever bear the sins and sorrows of the rest; but over the *blessedness* of his own spirit circumstance need have no control; God has therein given an unlimited power to the means of preservation, of grace and growth, at every man's command.—*J. H. Thom.*

---

"Joyful through hope," let us never lose sight of this, and in the darkness of the pit of trouble we shall see the stars shining above us, if we seem to have lost the light of the sun at noonday.—*Newbolt.*

### Echoes from the Outside World.

AN OPEN LETTER TO A HOSPITAL NURSE.

So much has been written and quoted by pro-Boers as to the fearful treatment meted out to the women and children in our concentration camps that a few facts gleaned from a Blue Book of nearly 400 pages, which has just been published, upon the working of these camps, will command the attention of everyone but the hopelessly prejudiced. There is not the slightest attempt made on the part of the authorities to deny that the mortality is exceedingly high, or that the food given out, though quite sufficient to keep even the nose of the wolf from the door, is not extravagantly liberal. But then there is no reason why it should be, and if concentration camps could be done away with altogether many think that it would be an advantage from every point of view. Up to the present time, with the exception of a bottle of milk a day for weakly children up to six years of age, the other articles of diet have been flour, coffee, salt, sugar, and meat. Now a pound a week of rice is to be added to the rations, and it is hoped that this may be beneficial in alleviating the diarrhœa and dysentery prevalent amongst the little folks. The high ratio of mortality is due partly to the camp life, which is very trying to young children, but largely to the insanitary habits of many of the Boer women. With a few exceptions they hate ventilation as much as they hate soap, and their habits are so far from cleanly that even with a rigid system of scavenging, the soil around the camp site becomes more insanitary every day. This has no doubt much to do with the virulence of the epidemic of measles which has broken out. The Boer mothers refuse to send the sufferers to the hospital, but let them run about with the rash thickly upon them; or if they are kept in bed for a day or two, get them up and feed them on meat directly the spots begin to diminish, so that pneumonia and peritonitis are frequently the fatal sequels to the disease itself.

November used in former years to be regarded as the month of fogs in England, but lately we have so frequently enjoyed open and bright weather during that particular period, that we had come to believe that some change had taken place in the meteorological conditions of this country. But, fearful lest it should lose its evil character, the penultimate month of the year has just given us examples of a genuine black fog such as have even surprised seasoned citizens of London. The climax was, I fancy, reached on Saturday evening. The cold was intense, twelve degrees of frost being registered in many parts. One omnibus driver was frozen to death on his box, and except here and there an impenetrable pall of darkness hung over all the metropolis. As early as half-past six an accident of rather a serious character occurred to a workmen's train between Brixton and Clapham, where 16 people were injured. Omnibus and cart catastrophes took place nearly all day, and cabs were often to be seen proceeding cautiously along the pavement which they were unable to distinguish from the road. One man took two hours to get from Mayfair to Baker Street, girls and boys with lanterns and candles earned many an honest penny by "showing the way," though it was often a case of the blind leading the blind, and dishonest rogues snatched at plunder and decamped without difficulty into the silent darkness with their booty. An old lady was seen walking about with a guttering candle in each hand, and the van from Nazareth House, going its round to collect food for the inmates, lost its way in Piccadilly, so that the driver found it impossible to get through the congested mass of vehicles. Nothing daunted, the Sister in charge alighted and led the horse. Then—so the story goes—three club young men saw her predicament, seized the horse's head, put the Sister inside the van, and took the cart safely to Nazareth House. They did not wait for thanks; but when someone, with a stronger belief in the supernatural than in the chivalry of man, suggested that perhaps the assistance had been rendered by angels, "No, no," said the Sister, "one was smoking a cigar!"

Some of you may have heard that Reuter's Telegram Company, like the District Messenger Company, have experienced a difficulty in obtaining a sufficient number of messengers of the male sex, and have therefore fallen back upon girls of 14 to 16 for the purpose. The secretary of Reuter's, after a three months' trial of the innovation, is disposed to be enthusiastic upon the working of the scheme. The girls, he says, are only employed in the day-time; they are supposed to be under strict control, and a check is kept upon their movements by a specific time only being allowed for each errand. On the other side "A Woman of Experience" writes a letter to the *Times*, pointing out that it is in every way bad training for young girls, and I must say, that from the feminine stand-point, her protest is well-timed and impressive. The modern girl from 12 to 13 of the class from which these messengers are drawn—and, alas! I must say in a higher class also, where more careful upbringing should be the rule—have only two desires in life. To leave school so as to be able to earn money to spend on pleasure and dress, and to be free to giggle and flirt with every boy and man they come across. The truth of this, with a few happy exceptions, will be apparent to all who have much to do with the half-grown girls of the present day, or who even use their eyes in the streets or parks on a summer evening or a Saturday half-holiday. Now a girl who wants to see "plenty of life," as she calls it, is often the very girl who is sharp enough and reliable enough to make a good messenger, but whose failings are daily fed by the many boys and men with whom she is brought into contact. Also "running errands" is not like an apprenticeship to a business which afterwards brings a return in steady work and good cash. When they are 17 or 18 these girls will become too old for their present situations, and their unrestrained life will make it hard for them to settle down to factory life or the workroom, much less the distasteful *rôle* of domestic servant. Mothers who are anxious that their daughters should earn money at once, and take no thought for the future, will let their girls become messengers; those who wish them to grow up a blessing to their own homes, or their husband's home later on, will find them, I fancy, another vocation in life.

Two of the London Galleries have opened their winter show of pictures. The Society of Portrait Painters contains several examples of the work of the veteran artist, Mr. Watts. How little Age has been able to rob his hand of its cunning is evident in a picture of "Miss Margery Dunthorne," which bears date of the present year, and pourtrays in a masterly style the pretty face of a young lady. The other canvases of Mr. Watts include "The Earl of Shrewsbury," and "The Marchioness of Northampton." Mr. Shannon wanders somewhat away from his beaten track and shows us that he can depict with as much skill the brightness and freshness of a young boy, "Gerard, son of A. Thornton, Esq.," as the femininity and the elaborate raiment of a "Lady with a Chinese Fan." Several of the foreign contributors stand out conspicuously in the exhibition. Herr Franz von Leubach shows a noble portrait of "H.M. the late Emperor Frederick" —which of course is not new—as also a picture of a lady with ruddy-coloured hair and a strangely artificial smile, and two portraits of the artist and his family. M. Lucien Joseph Simon—who is little, if at all, known in England—has painted a very striking picture entitled "The Portrait of an Old Woman," which is lifelike and exceedingly clever. At the Royal Society of British Artists there is a pleasant, if not a particularly strong, collection. Mr. Windsor Fry has been very successful in his character sketch of "Hamlet." The young man's face is pale and haggard, framed in auburn-coloured hair, and the subtle combination of madness and genius in the eyes has been cleverly emphasised. Mr. T. M. Sheard shows a thorough knowledge of rustic life and surroundings in "Parted"—two old villagers by the fire, the man's hand laid upon his wife's, and sorrow clearly marked upon the two faces. "Sunny Noon," by Mr. John McIntosh, is refreshingly bright and realistic, especially in this foggy month, and "Staging in California," is a stirring piece of work, instinct with movement.

### Notes and Queries.

**The Editor is always willing to answer in this column, without any fee, all reasonable questions, as soon as possible.**
**But the following rules must be carefully observed :—**
**1. Every communication must be accompanied by the name and address of the writer.**
**2. The question must always bear upon nursing, directly or indirectly.**
**If an answer is required by letter a fee of half-a-crown must be enclosed with the note containing the inquiry.**

*Publications.*

(78) Who are the publishers of Jerneux's "Physiology," and what is the price? Is it a good book for an intending probationer to study; or is there any other which you consider better?—*C. M. E.*

Huxley's "Physiology" is one which is commonly used.

*Home Nursing.*

(79) I should be grateful if you can tell me if there is any course of instruction I can go through with a view to fitting myself to give lectures on Home Nursing either for the School Board or anywhere I could obtain employment. The curriculum of the National Health Society is too expensive, and it is more the actual lecturing I wish to prepare for than the subject. I have been nursing for over ten years, and should have a fair knowledge of that.—*C. M. C.*

If it is a question of the actual lecturing, the art of speaking and expressing oneself, rather than the knowledge of the subject itself, your best plan would probably be to apply to one or other of the various teachers of elocution. Our impression is, however, that, if you know your subject well, attendance at a few lectures would give you a good idea of the way in which the subject may be attacked, and then you might work it out in your own way.

*Hot Bath during Menstruation.*

(80) Could you kindly tell me if it is harmful to take a hot bath during menstruation? I have read in some of the best midwifery books that it is not, and in others that it is. One of my patients has tried it, and it has not done any harm.—*E. W.*

If a person is in the habit of taking a warm bath regularly, there is no reason, supposing menstruation to be normal, for such a habit being disturbed by the onset of that process. But what is called a *hot* bath, especially in the case of a person unaccustomed to it, causes a great disturbance of the circulation, and although we should greatly question its having any particular effect upon a case of absolutely normal menstruation, it must be remembered that with a large proportion of modern women, menstruation is not absolutely normal, and that where there is any tendency for the discharge to cease prematurely, or to "break off," and then return again, the taking of a hot bath, and the consequent diversion of a large proportion of the blood flow skin-wards might easily upset the apparently normal progress of events. One has to go by large numbers. Individuals may do things without injury, which to others would be injurious, and one so often finds menstruation upset by trivial causes that, without in any way saying that a hot bath would be injurious to a perfectly healthy woman during the course of a perfectly normal process, it is evident that taking the average there are risks in such a proceeding.

*Enema.*

(81) Can you tell me the proper proportions and best method of making starch mucilage for an enema? I have mixed the starch to a paste with cold water, and added boiling water, stirring well, but the starch does not clear. Should the boiling water be added quickly or slowly? It has been suggested to me that the fault lies in the quality of the starch, but I think it is rather in the proportions or in the method of mixing.—*Non Capisco.*

The following are the directions given in the British Pharmacopœia for the preparation of mucilage of starch. "Triturate 1 gramme of starch with a small quantity of distilled water to form a smooth paste; add more distilled water, gradually, to produce 50 cubic centimetres of mixture; boil for a few minutes, constantly stirring; cool. Mucilage of starch should be recently prepared." The Guy's Hospital Pharmacopœia gives much the same directions, but states the quantities in English weights and measures, as 120 grains of starch and 10 fluid ounces of water. Of course, this mucilage "sets" when it becomes cold. About 2 ounces is the quantity commonly employed as a basis for an enema of opium. It becomes fluid again when warmed to the temperature of the body.

*Phagocytes.*

(82) Will you kindly tell me where I can learn about "Phagocytes," the organisms in our bodies which repel disease? I am greatly interested in the subject.—"*Pat.*"

In any treatise on bacteriology, or in Vol. I. of "Allbutt's System of Medicine."

*Medical Studies.*

(83) Do you know of any college or society in which a young man of good education could pursue medical studies free?—*Nurse X. Y. Z.*

No. Unless he got a scholarship.

*Brighton Homes.*

(84) Will you kindly tell me if there are any homes in Brighton suitable for a patient who is commencing with general paralysis? The patient is a lady about 42, and unable to make any, or only a very small, payment.—*Nurse E.*

We know of no suitable public institution, and we cannot recommend private homes. The disorder which is usually meant by the term "general paralysis" is a disorder of the brain which in most cases sooner or later requires to be dealt with in an asylum.

*Probationers.*

(85) I wish to be trained as a hospital nurse and should be glad to know if a hammer toe would prevent my being accepted by a good London Hospital.—*K. C.*

If the defect does not interfere with your standing and walking it should not prevent you from becoming a nurse. Consult a medical man.

I hold a certificate of 2 years and 10 months from a children's hospital and I have entered this fever hospital for two years. I now find that this qualification is not equal to that of a three years' training in a general hospital. Will you tell me what I had better do? I don't want to begin as probationer again after completing my two years here. I am a Catholic, and wish to obtain work in a Catholic institution.—*Impulsive One.*

To gain a three years' certificate you must begin to train as a probationer in a general hospital upon the completion of your present engagement; or you may make the fever nursing of children a speciality.

I am anxious to go as hospital nurse at once. Can you kindly tell me at which hospital to apply? I shall be twenty in November.—*L. L.*

You cannot become a hospital nurse until you are twenty-three, because no good general hospital will train you before you attain that age. See the "Nursing Profession: How and Where to Train," for children's hospitals, which accept probationers at 20.

1. Can you tell me where to obtain particulars of American nursing schools? 2. Are nurses well paid in America? 3. Would an American hospital be likely to take me without seeing me first?—*H. B. W.*

1. You will find a valuable list of American training schools in the "Nursing Profession: How and Where to Train." 2. The pay for nurses is good in America, but the expenses are correspondingly heavy. 3. This is very doubtful.

I wish to train as a nurse, but my sight though good is short. Will you kindly tell me if wearing spectacles would disqualify me?—*E. N. P.*

The medical officer examining the probationers for the institution which accepts you will answer this.

1. Will you kindly tell me how to obtain a position as a probationer in any hospital in South Africa? 2. Where can I apply for information concerning hospital appointments abroad?—*M. P.*

1. There are training schools for nurses attached to the Johannesburg Hospital (P.O. Box 1050) and to the Provincial Hospital, Port Elizabeth, Cape Colony, but applications for training are many and vacancies few. You would be well advised to train in England. 2. The Colonial Nursing Association, Imperial Institute, W.

Will you kindly tell me what book would be a help in preparing for the entrance examination for probationers at a London hospital? Is the examination difficult?—*General Reader.*

You only require to pass in elementary subjects—reading, writing and arithmetic—which any well-educated, intelligent girl can easily do.

*A Question of Arithmetic.*

(86) Will you kindly tell me the quantity of boracic acid crystals to use to a pint of water to make a solution of 1 in 30?—*J. D.*

This is a mere question in arithmetic, but owing to the insane weights and measures in use in England it is a somewhat complicated one. There are 70,000 grains of water in a gallon, and a pint is an eighth part of a gallon. Hence there are 8,750 grains of water in a pint. Divide this by 30 and one has $291\frac{2}{3}$ grains of boracic acid to make a pint of 1 in 30. Nine people out of ten, however, would regard minims as equal to grains, and as in a pint there are $20 \times 480$ minims they would divide this by 30 and get $\frac{20 \times 480}{30} = 320$ grains.

### Standard Books of Reference.

"The Nursing Profession: How and Where to Train." 2s. net; post free 2s. 4d.
"Burdett's Official Nursing Directory." 3s. net; post free, 3s. 4d.
"Burdett's Hospitals and Charities." 5s.
"The Nurses' Dictionary of Medical Terms." 2s.
"Burdett's Series of Nursing Text-Books." 1s. each.
"A Handbook for Nurses." (Illustrated). 5s.
"Nursing: Its Theory and Practice." New Edition. 3s. 6d.
"Helps in Sickness and to Health." Fifteenth Thousand. 5s.
"The Physiological Feeding of Infants." 1s.
"The Physiological Nursery Chart." 1s.; post free, 1s. 3d.
"Hospital Expenditure: The Commissariat." 2s. 6d.
All these are published by the Scientific Press, Ltd., and may be obtained through any bookseller or direct from the publishers 28 and 29 Southampton Street, London, W.C.

### Travel Notes.

By Our Travelling Correspondent.

### LXXXVI.—LITTLE KNOWN SPOTS ON THE RIVIERA—PEILLE—PEILLON, ETC.

Peille, which is called on some maps Peglia, is a delightful excursion for those with good walking powers, or cyclists. There are two or three ways of reaching these mountain villages.

Let us first take Peille, which is to my taste the cream of the group. This can be done either from Mentone or Nice; from Mentone you go by the Gorbio road, and it is much nearer than viâ Nice but not so fascinating. If you are at Nice you can help yourself a long way on the road by taking the diligence that goes to Tenda, and leaving it at the Pont de Peille; by this means you have shortened your walk by seven and a half miles, and there will remain about four miles only to walk. If you cycle you will go through Trinité Victor and Drap, arriving at the Plan de Peille; here you must leave your machines and make the ascent on foot. The mule tracks are rather confusing, but they all lead in the same direction straight up to Peille. From Nice to the Plan de Peille it will take you an hour and a half's easy wheeling, but the road is so lovely that yon will probably take much longer to do it. Start very early in the morning if you can so as to evade the heat and to have a long day before you. There is no reason against sleeping at Peille if you like; there is a clean and comfortable little inn called the Hôtel Mont Boron, from the majestic mountain rising opposite.

The village is of the usual kind, a stronghold built at a great altitude to be safe from marauders of all kinds, and especially the Moorish pirates, who as lately as the end of the eighteenth century sometimes carried off natives of the Rivieras for slaves. The streets are so narrow that those carrying burdens on their heads must manœuvre a little if they meet anyone. As no wheeled traffic can arrive there it was naturally thought unnecessary to make wide streets. The effect of these narrow alleys is very picturesque, and in sketching there is such a wealth to choose from that one does not know where to begin. Houses are built on archways spanning the narrow streets, others hang precariously on the hill side, and almost all are adorned with clothes to dry, many out on long poles projecting from the neighbourhood of windows; this primitive arrangement seems to me peculiar to old Provence. There is a square, indeed there are two, one where the inn is, and the other quite pretentious, with a fountain and municipal buildings. I spent two nights at Peille, and could have stayed there very happily a week, but few have time for this leisurely travelling, and one long day will afford you immense pleasure.

### Peillon.

For a day at Peillon, take the diligence or cycle as far as the Pont de Peille, there you turn off to the right still keeping to your cycle; indeed you can ride nearly all the way till you come to some oil mills, where you can leave your cycle and get something refreshing to drink. From there the climb will take you half an hour. The village is not so picturesque as Peille, but the last half of the road being different and very impressive, it will form an interesting day's outing. There is a curious old church with some very primitive decorative work and pictures of little merit. Should you decide to cycle all the way the distance from Nice to the oil mills is between eight and nine miles, and to Peille about twelve. So neither excursion is really a serious undertaking.

### Biôt.

This is a very easy excursion either from Nice or Cannes, or even Grasse. From the station of Antibes there is a diligence that goes straight into Biôt. If you are staying at Grasse take the train to Cannes and there either change trains to Antibes and thence by diligence or go by cycle; this latter way is the nicest. There is no other road that I am aware of from the Cannes side beyond that turning off from Antibes, and the distance from the station would be three miles or perhaps not more than two and a half. If you start from Nice you would cycle or take the train to Var station, where you turn off for Biôt *via* Cagnes; this is of course a much longer journey, about 14 miles altogether, but the route is very fine, and there are some remarkable views at different points.

Biôt itself is a humble little place, but it has rather an interesting history, for it was once in the possession of the Knights of Malta and also of the Knights Templars. You will have time in the same day to visit Antibes, which, being on the main Riviera, is well known now, but it is a strange and still primitive place. The effect of its massive and brilliantly coloured towers against the intensely blue sky is very beautiful. The entrance to this old-world town, the Antipolis of the ancients, is through a fine gateway and over a fosse. The ramparts are grand and entirely surround the town; once the place must have been impregnable. Close to the church, which is rather beyond the average in interest, we come upon the two towers, which arrest one's attention in all views of Antibes.

Vallauris, where they make such pretty pottery, is near Cannes, whence an omnibus makes the journey many times a day.

### Les Iles des Lerins.

An interesting and non-fatiguing excursion is to the Iles des Lerins. Of the two St. Honorat is really the more interesting, but St. Marguerite perhaps takes the fancy more, because in the reign of Louis XIV. the unfortunate prisoner with the iron mask was confined there for weary months before his removal to the Bastille. The mystery surrounding his identity has lately been made quite clear, with the attendant loss of some romance, and the memory of the Grand Monarque has not achieved fresh lustre in the process. There are steamers that ply constantly from Cannes, taking about half an hour to reach St. Marguerite, and 40 minutes to St. Honorat. The latter is a mine of antiquarian interest. Until the great Revolution of 1793 seven chapels stood round the shore, now only three remain in a good condition.

The castle of Lerins is immediately on the shore, almost with its feet in the water, and there is much to be seen by the archæological explorer.

### TRAVEL NOTES AND QUERIES.

Rules in Regard to Correspondence for this Section.—All questioners must use a pseudonym for publication, but the communication must also bear the writer's own name and address as well, which will be regarded as confidential. All such communications to be addressed "Travel Editor, 'Nursing Mirror,' 28 Southampton Street, Strand." No charge will be made for inserting and answering questions in the inquiry column, and all will be answered in rotation as space permits. If an answer by letter is required, a stamped and addressed envelope must be enclosed, together with 2s. 6d., which fee will be devoted to the objects of "The Hospital" Convalescent Fund. Any inquiries reaching the office after Monday cannot be answered in "The Hospital" of the current week.

Bayeux and Vicinity (Sister Lilian).—Sorry this question was overlooked. You can go *viâ* Southampton and Cherbourg, second-class return, £2 2s. 4d., at all seasons, but the cheaper and more direct way is *viâ* Newhaven and direct steamer to Caen, second-class return, £1 5s., and the 20 miles on to Bayeux by rail would be, second-class return, about 5s. This service of boats only runs, however, during the summer months. Bayeux is four miles inland. The neighbourhood teems with interest; within excursion distance lies Caen, infinitely more interesting than Bayeux and Lisieux, which is a mine of artistic interest. Twelve miles north on the Calvados coast is the charming little village of Arromanches. Let me hear if I can help you further.

